# Effects of Tai Chi Chuan on Postural Stability and Lower-Limb Biomechanical Characteristics in Patients With Functional Ankle Instability: A Randomized Controlled Trial

**DOI:** 10.1016/j.arrct.2026.100595

**Published:** 2026-02-04

**Authors:** Chen Zhang, Dunbing Huang, Zhonghua Lin, Jiaqi Wang, Hongfei Ren, Lihong Li, Xiaohua Ke, Zhenhua Wu, Cai Jiang

**Affiliations:** a Rehabilitation Medicine Department, The Third Affiliated Hospital of Zhejiang Chinese Medical University, Hangzhou, Zhejiang.; b Encephalopathy and Rehabilitation Center, The Second Affiliated Hospital of Zhejiang Chinese Medical University, Hangzhou, Zhejiang.; c Rehabilitation Medicine Center, Fuzhou University Affiliated Provincial Hospital, Fuzhou.; d Department of Rehabilitation Medicine, Shengli Clinical Medical College of Fujian Medical University, Fuzhou.; e Department of Rehabilitation Medicine, Shanghai Fourth People’s Hospital, School of Medicine, Tongji University, Hongkou, Shanghai, China.

**Keywords:** Balance function, Biomechanics, Functional ankle instability, Gait analysis, Rehabilitation, TCC

## Abstract

**Objectives:**

To evaluate the effects of a 12-week Tai Chi Chuan (TCC) program on ankle instability/function, pain, and gait/balance performance in patients with functional ankle instability (FAI).

**Design:**

Prospective, single-blind randomized controlled trial.

**Setting:**

Clinical gait analysis laboratory with community-based recruitment.

**Participants:**

Fifty patients with FAI were randomly divided 1:1 to a TCC group (n=25; age 65.44±8.75y; 18 women/7 men) or a control group (n=25; age 67.08±4.76y; 22 women/3 men). By eligibility criteria, all participants had experienced at least 1 severe ankle sprain within the prior year and had recurrent episodes of giving way/instability in the prior year.

**Interventions:**

The TCC group completed supervised simplified 24-form TCC training 3 sessions/wk for 12 weeks; controls received structured health education once every 4 weeks for 12 weeks.

**Main Outcome Measures:**

The primary outcome was the Cumberland Ankle Instability Tool (CAIT). Secondary outcomes included the American Orthopaedic Foot & Ankle Society Ankle–Hindfoot Score, pain intensity (visual analog scale [VAS]), 3-dimensional gait spatiotemporal and sagittal kinematic parameters, and postural control (Huber360 static balance metrics and the Y-Balance Test [YBT]).

**Results:**

After 12 weeks, CAIT increased by 10.32 points in the TCC group (14.48±5.53 to 24.80±4.21) compared with 2.84 points in the control group (15.16±5.98 to 18.00±5.05), with a significant between-group difference (*P*<.01), exceeding the CAIT minimal clinically important difference (MCID) (≥3 points); American Orthopaedic Foot & Ankle Society Ankle–Hindfoot Score and VAS also improved more with TCC (all *P*<.05), with changes within/above published AOFAS MCID estimates (7.9-30.2 points) and exceeding the commonly used VAS MCID (∼1.4 cm). The TCC group also demonstrated improvements in step width, selected sagittal-plane hip/ankle kinematics, and Y-Balance Test performance compared with controls (*P*<.05).

**Conclusions:**

A 12-week TCC program may provide clinically meaningful improvements in ankle stability and function, reduce pain, and enhance gait and balance performance in patients with FAI.

Ankle sprain is among the most common lower-extremity injuries, and a substantial proportion of individuals develop persistent symptoms after the initial injury.^1^ Recurrent sprains and incomplete recovery can lead to chronic ankle instability, characterized by pain, episodes of giving way, and impaired sensorimotor control.^2^ Functional ankle instability (FAI) refers to recurrent instability symptoms without clear mechanical laxity and is clinically important because it may increase reinjury risk and contribute to altered movement control beyond the ankle.^3^^,^^4^

Standard rehabilitation for FAI emphasizes strengthening, proprioceptive training, and balance exercises; however, residual deficits and adherence challenges remain common, particularly in individuals with pain or fear of reinjury.^5^^,^^6^ Safe, engaging, and sustainable interventions that target neuromuscular control and postural stability are therefore needed.

Tai Chi Chuan (TCC) is a low-impact mind–body exercise involving slow, controlled weight shifting, single-leg support, and coordinated multijoint movements. As a structured modality, it emphasizes smooth transitions and alignment and may improve proprioception, balance, and motor control.^7^ These features are relevant to FAI, which involves peripheral neuromuscular deficits and altered sensorimotor integration. However, evidence in FAI is limited and largely patient-reported, leaving uncertainty about effects on objective gait and balance.

Objective assessment using 3-dimensional gait analysis and instrumented postural control testing may provide a more comprehensive evaluation of functional changes after TCC training.^8^^,^^9^ Therefore, this single-blind randomized controlled trial investigated the effects of a 12-week TCC program on ankle instability/function and pain and on gait and balance in patients with FAI. We hypothesized that, compared with health education, participants receiving TCC would demonstrate greater improvements in ankle instability/function and pain and show more favorable gait and balance performance, reflected by improvements in spatiotemporal parameters, sagittal-plane lower-limb kinematics, and postural control measures.

## Methods

### Study design

**Fig 1:**
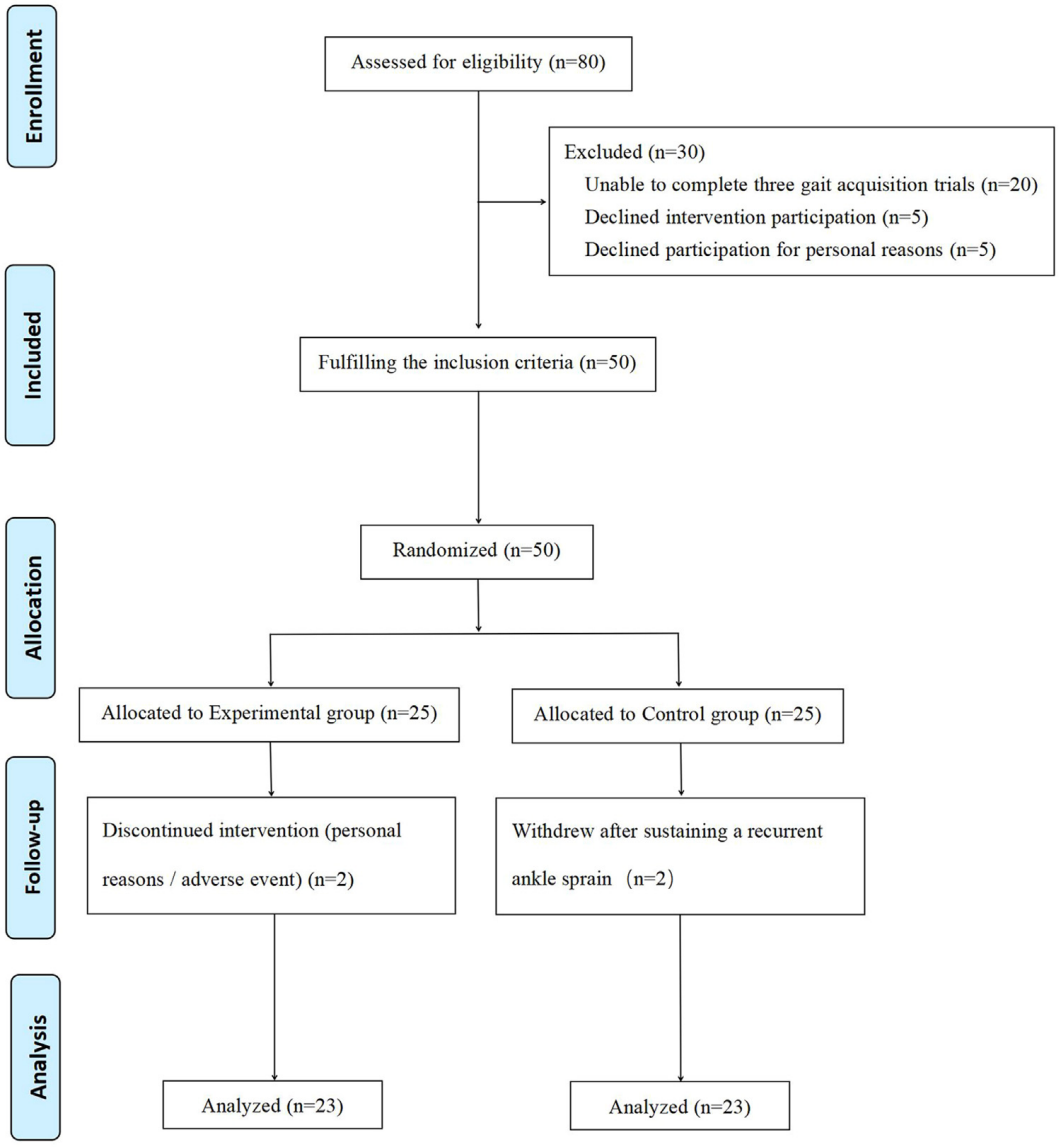
CONSORT flow diagram of participant screening, eligibility, randomization, follow-up, and analysis.

This prospective, single-blind randomized controlled trial compared a supervised 12-week TCC program with a health education control in adults with FAI. The protocol was approved by the Ethics Committee of Fujian Provincial Hospital (K2019-03-035), registered with the Chinese Clinical Trial Registry (ChiCTR2100041790; March 22, 2021), and all participants provided written informed consent. Participant flow is summarized in figure 1.

### Sample size

Sample size was estimated using G*Power^a^ based on Cumberland Ankle Instability Tool (CAIT) as the primary outcome, using effect estimates from Xu et al^10^ (effect size d=0.97). With α=0.05 and 85% power, 24 participants per group were required. Allowing for 20% attrition, we aimed to enroll 25 participants per group (N=50). This trial was powered to detect a between-group difference in CAIT. The sample size was not powered for American Orthopaedic Foot & Ankle Society Ankle–Hindfoot Score (AOFAS-AHS) or the visual analog scale (VAS), which were analyzed as secondary clinical outcomes.

### Participants

Participants were recruited from hospitals, community centers, and sports academies via advertisements. Eligible adults (≥18y) had right-dominant FAI defined as: at least 1 severe ankle sprain within the past year resulting in ≥1 day of functional limitation; recurrent symptoms (≥2 episodes of giving way and/or perceived unilateral ankle instability within the past year); and a CAIT score ≤24.^11^ Participants were additionally required to have no history of sprain or instability symptoms in the contralateral ankle and no prior surgery or suspected mechanical instability of the affected ankle. Exclusion criteria included prior ankle/lower-limb fracture or surgery, acute lower-limb injury that could affect testing, neurologic/neuromuscular or vestibular conditions associated with balance dysfunction, or visual impairment preventing participation in balance assessments.

### Randomization and blinding

All eligibility criteria were confirmed before randomization, and only eligible participants proceeded to allocation. Randomization was performed in a 1:1 ratio using a computer-generated sequence in SPSS^b^. Allocation was concealed using opaque, sequentially numbered sealed envelopes opened at enrollment, assigning participants to the experimental or control group. Because of the nature of the intervention, instructors and participants were not blinded; however, outcome assessors, data collectors, and statisticians were blinded to group allocation.

### Interventions

#### Control group

The control condition was intended to represent usual-care management and served as a minimal-intervention comparator. Participants received structured health education once every 4 weeks for 12 weeks (three 30-min sessions), covering ankle sprain prevention and self-management (activity modification and appropriate rest), guidance on avoiding symptom-provoking activities, use of ankle bracing when needed, basic stress-management/psychological support, and general dietary advice.

#### Experimental group

Participants in the experimental group completed a supervised 12-week simplified 24-form TCC program delivered by an experienced instructor who was not involved in outcome assessment or data analysis. This intervention was designed to test the incremental benefit of supervised TCC beyond usual-care education; therefore, no additional structured education sessions were delivered as part of the experimental intervention.

Each session comprised a 10-minute warm-up, 30-minute simplified 24-form TCC practice, and 10-minute cool-down/relaxation. Weeks 1-4 focused on learning technique, whereas weeks 5-12 emphasized consolidation and progressive practice (eg, direction changes and repeated bouts with brief recovery). Sessions were held 3 times per wk for 12 weeks (36 sessions). Heart rate was monitored during each session using an ALPHA 2 heart rate monitor^c^ to verify training within the prespecified safe range.

A schematic overview of the 24-form routine is provided in supplemental figure S1 (available online only at http://www.archives-pmr.org/).

### Outcome measurements

Two trained evaluators, blinded to group allocation, performed all assessments within 3 days before the intervention and within 3 days after completion.

#### Primary outcome measures

Foot and ankle function: the CAIT is a self-reported measure of perceived ankle instability (0-30; higher scores indicate better stability); a score ≤24 is commonly used to indicate FAI. The CAIT has demonstrated good reliability and validity^12^ and the reported minimal clinically important difference (MCID) (and minimal detectable change) is ≥3 points.^13^

#### Secondary outcome measures

Ankle–hindfoot function: the AOFAS-AHS (0-100) comprises pain (0-40), function (0-50), and alignment (0-10) domains and has demonstrated acceptable reliability and construct validity in foot/ankle populations.^14^ It includes both patient-reported items and clinician-assessed components; in this trial, it was completed by a blinded evaluator using a standardized interview and physical examination.^14^ Because AOFAS MCID values are diagnosis and context specific, we report published MCID estimates from hallux valgus surgery (7.9-30.2 points) as a reference when interpreting clinical relevance.^15^

Pain: pain intensity was measured using a 10-cm horizontal VAS (0=no pain, 10=worst imaginable pain).^16^ Participants rated their average ankle pain during daily activities over the past week, and the distance (cm) from the 0 anchor was recorded. A commonly used MCID threshold for musculoskeletal pain on a 10-cm VAS is approximately 1.4 cm.^17^

### *Three-dimensional gait analysis*

**Fig 2:**
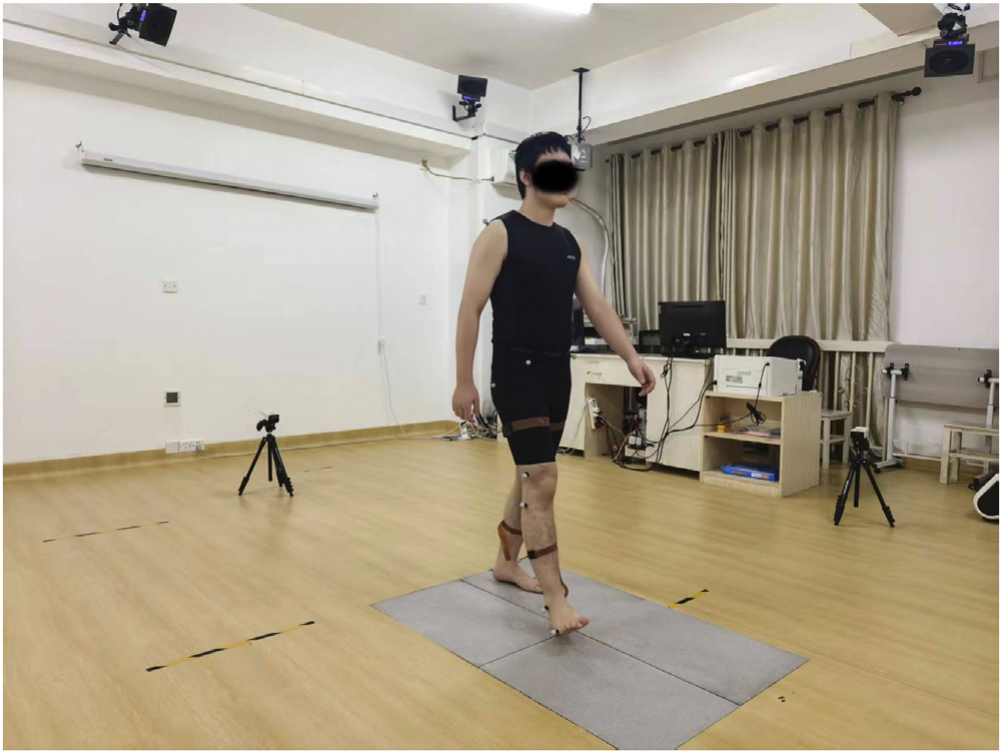
Laboratory setup and reflective marker placement for 3-dimensional gait analysis.

Gait data were collected using the SMART-D 400 infrared motion capture system^d^ with 8 cameras sampling at 50 Hz, synchronized with embedded P6000D force plates. The capture volume was calibrated before each session (manufacturer static/dynamic procedures), and the laboratory coordinate system was defined as X anterior (walking direction), Y mediolateral, and Z vertical. Eighteen 14-mm reflective markers were placed bilaterally at the anterior superior iliac spine, posterior superior iliac spine, lateral thigh (rigid cluster), lateral femoral epicondyle, lateral shank (rigid cluster), lateral malleolus, posterior calcaneus, and the first and fifth metatarsal heads; cluster markers were secured with elastic wraps and the remaining markers with hypoallergenic double-sided tape. The laboratory setup and reflective marker placement are shown in figure 2.

Participants walked barefoot along an 8-m walkway at a self-selected comfortable speed for 5 passes. Force plates were located mid-walkway and participants were instructed not to target them. Heel strike and toe-off were identified using vertical ground reaction force thresholds for valid contacts; otherwise, heel/toe marker kinematics (position/velocity) were used. Spatiotemporal and kinematic outcomes were summarized from 5 central gait cycles per participant. Step length and step width were calculated as the anteroposterior (X) and mediolateral (Y) distances, respectively, between heel markers at contralateral heel strike. Cadence (steps/min) and walking speed (from forward pelvic displacement over time) were calculated across analyzed cycles.

Marker trajectories were processed using BTS SMART software^d^, visually inspected, and briefly interpolated when feasible; trials with excessive marker loss were excluded. Trajectories were low-pass filtered (zero-lag 4th-order Butterworth, 6 Hz), a cutoff commonly used for level walking marker trajectories in optical gait analysis.^18^ Segment coordinate systems (pelvis, thigh, shank, foot) were defined from the marker set, and hip/knee/ankle angles were computed using a Cardan XYZ sequence (X flexion/extension, Y abduction/adduction, Z internal/external rotation) consistent with International Society of Biomechanics recommendations for reporting joint motion^19^ and the joint coordinate system convention.^20^ We did not perform formal sensitivity analyses across alternative cutoffs or rotation sequences; however, processing parameters were prespecified and waveforms were visually inspected for smoothness and plausibility. We report sagittal-plane angles (hip/knee flexion–extension; ankle dorsiflexion–plantarflexion), including peak values (maximum/minimum across 0%-100% of the gait cycle) and angles at heel strike (initial contact); for the ankle, the maximum indicates peak dorsiflexion and the minimum indicates peak plantarflexion.

Because frontal- and transverse-plane alterations have been reported in chronic ankle instability/FAI, we acknowledge that multiplanar kinematics may provide additional insight. However, to limit multiple comparisons and because sagittal-plane measures during level walking are generally more robust to soft-tissue artifact in older adults, we a priori focused our analysis and reporting on sagittal-plane kinematics.

#### Postural control ability

Static postural stability was assessed using the Huber^e^ during single-leg stance under eyes open and eyes-closed conditions (50s each). Each participant completed 1 familiarization trial (not analyzed). Outcomes included center of pressure speed (mm/s), trajectory length (mm), and trajectory area (mm^2^); higher values indicate poorer stability.

Dynamic stability was assessed using the Y-Balance Test (YBT). After 1 familiarization session, participants stood on the affected limb with hands on the iliac crests and performed maximal reach trials with the contralateral limb in 3 directions: anterior, posteromedial (45°), and posterolateral (45°). Three trials were recorded per direction with 1-minute rest; values were averaged by direction. The composite score was calculated as: Composite (%)=(anterior + posteromedial + posterolateral)/(3 × limb length) × 100.^21^

### Statistical analysis

Data analysis was performed using SPSS^b^. Continuous variables were expressed as mean±SD (normal) or median (interquartile range) (non-normal), and categorical variables as frequencies. Between-group comparisons used independent-sample *t* tests or Mann-Whitney *U* tests; within-group comparisons used paired *t* tests. Categorical data were analyzed using the chi-square test. A 2-sided *P* value <.05 was considered statistically significant.

## Results

### Baseline characteristics

Baseline characteristics were comparable between groups (table 1). Of 50 randomly divided participants (25 per group), 46 completed the 12-week follow-up and were included in the pre–post analyses (n=23/group). There were no significant between-group differences at baseline in age, sex, body mass index, or patient-reported ankle instability/function and pain (CAIT, AOFAS-AHS, and VAS; all *P*>.05).

**Table 1:** Baseline demographic and clinical characteristics of the participants.

Demographic Characteristic	Experimental Group (n=25)	Control Group (n=25)	t/χ^2^	*P*
Age (y)	65.44±8.75	67.08±4.76	0.82	.41
Sex			28.00	.26
Male	7	3		
Female	18	22		
BMI (kg/m^2^)	23.98±2.96	24.21±2.61		
CAIT	14.48±5.53	15.16±5.98	−0.67	.50
AOFAS	77.68±9.61	77.08±9.20	−0.23	.82
VAS	3.2±2.20	3.00±2.58	−0.59	.56

Abbreviation: BMI, body mass index.

### Foot and ankle function

After 12 weeks, the TCC group demonstrated greater improvements in ankle function and pain than controls (table 2). CAIT increased by 10.32 points (14.48±5.53-24.80±4.21) versus 2.84 points (15.16±5.98-18.00±5.05) (*P*<.01), exceeding the reported CAIT MCID (≥3 points). AOFAS-AHS increased by 15.16 points (77.68±9.61-92.84±8.11) versus 4.24 points (77.08±9.80-81.32±8.80) (*P*<.01). VAS decreased by 1.84 cm (3.20±2.20-1.36±1.93) versus 1.04 cm (3.00±2.58-1.96±1.54) (*P*=.03), and the mean reduction in the TCC group exceeded the commonly used VAS MCID (∼1.4 cm).

**Table 2:** Ankle function score at baseline and 12 weeks in the experimental and control groups (mean±SD).

	Experimental Group (n=23)	Control Group (n=23)	Between-Group Difference
CAIT			
Baseline	14.48±5.53	15.16±5.98	0.09 (0.46-0.55), *P*=.50
12 wk	24.80±4.21	18.00±5.05	0.61 (0.00-0.00), *P*<.01*
Within-group change (12 wk)	−0.58 (0.00-0.00), *P*<.01*	−0.27 (0.036-0.072), *P*<.01*	
AOFAS			
Baseline	77.68±9.61	77.08±9.20	−0.06 (−0.62 to 0.49), *P*=.82
12 wk	92.84±8.11	81.32±8.80	0.58 (0.00-0.00), *P*<.01*
Within-group change (12 wk)	−0.53 (0.00-0.01), *P*<.01*	−0.26 (0.042-0.082), *P*=.08	
VAS			
Baseline	3.20±2.20	3.00±2.58	0.08 (0.54-0.62), *P*=.56
12 wk	1.36±1.93	1.96±1.54	0.31 (0.02-0.05), *P*=.03*
Within-group change (12 wk)	−0.36 (0.00-0.01), *P*=.01*	−0.34 (0.012-0.038), *P*=.02*	

⁎ indicates that there is a statistically significant difference between groups, *P*<.05.

### Spatiotemporal parameters

Spatiotemporal gait parameters were similar between groups at baseline (table 3). At 12 weeks, step width decreased in the TCC group (−0.02m) but increased slightly in controls (+0.01m), resulting in a significant between-group difference (*P*=.04). No other spatiotemporal variables differed.

**Table 3:** Spatial and temporal parameters at baseline and 12 weeks in the experimental and control groups (mean±SD)

	Experimental Group (n=23)	Control Group (n=23)	Between-Group Difference
Stride length			
Baseline	1.07±0.10	1.1±0.14	0.27 (−0.29 to 0.82)*, P=*.35
12 wk	1.10±0.10	1.1±0.16	−0.05 (−0.61 to 0.5)*, P=*.85
Within-group change (12 wk)	−0.29 (−0.68 to 0.12)*, P=*.17	0.04 (−0.36 to 0.43)*, P=*.85	
Stance phase			
Baseline	59.64±7.89	61.13±2.79	−0.09 (0.52-0.58)*, P=*.52
12 wk	60.41±1.99	60.65±5.35	−0.19 (0.179-0.229)*, P=*.18
Within-group change (12 wk)	−0.1 (0.45-0.51)*, P=*.46	−0.03 (0.82-0.84)*, P=*.82	
Swing phase			
Baseline	37.82±5.85	38.87±2.77	−0.23 (0.09-0.13)*, P=*.11
12 wk	39.54±2.03	38.07±3.48	−0.52 (−1.08 to 0.05)*, P=*.08
Within-group change (12 wk)	−0.25 (0.06-0.1)*, P=*.08	0.19 (−0.2 to 0.59)*, P=*.34	
Speed			
Baseline	0.99±0.19	1.00±0.16	0.02 (−0.53 to 0.58)*, P=*.94
12 wk	1.03±0.12	1.02±0.18	−0.05 (−0.61 to 0.5)*, P=*.85
Within-group change (12 wk)	−0.19 (−0.58 to 0.21)*, P=*.36	−0.14 (−0.53 to 0.26)*, P=*.51	
Cadence			
Baseline	108.97±11.65	108.82±7.75	−0.02 (−0.57 to 0.54)*, P=*.96
12 wk	112.70±8.71	106.30±20.82	−0.17 (0.202-0.254)*, P=*.23
Within-group change (12 wk)	−0.26 (−0.65 to 0.14)*, P=*.21	−0.03 (0.86-0.88)*, P=*.85	
Step length			
Baseline	0.49±0.1	0.51±0.08	−0.08 (0.52-0.59)*, P=*.55
12 wk	0.54±0.08	0.57±0.10	−0.11 (0.42-0.48)*, P=*.44
Within-group change (12 wk)	−0.30 (0.03-0.05)*, P=*.04*	−0.36 (0.01-0.01)*, P=*.01*	
Stride width			
Baseline	0.10±0.03	0.09±0.04	−0.17 (0.22-0.28)*, P=*.23
12 wk	0.08±0.03	0.10±0.04	−0.30 (0.03-0.054)*, P=*.04*
Within-group change (12 wk)	0.51 (0.00-0.00)*, P<*.00*	−0.17 (−0.56 to 0.23)*, P=*.41	

⁎ indicates that there is a statistically significant difference between groups, *P*<.05.

### Gait sagittal kinematics

Baseline sagittal-plane kinematics were comparable between groups (table 4). At 12 weeks, compared with controls, the TCC group showed a lower hip flexion maximum (41.51±6.28 vs 42.84±5.68, *P*=.04) and greater hip flexion at heel strike (38.96±4.83 vs 36.13±3.57, *P*=.02). The TCC group also had lower peak ankle dorsiflexion (16.18±3.39 vs 18.86±4.90, *P*=.03) and lower ankle dorsiflexion at heel strike (11.02±2.53 vs 12.75±3.41, *P*=.04).

**Table 4:** Comparison of sagittal kinematic parameters at baseline and 12 weeks in the experimental and control groups (mean±SD)

	Experimental Group (n=23)	Control Group (n=23)	Between-Group Difference
Hip flex-extension MAX			
Baseline	44.94±13	43.52±5.67	−0.1 (0.47-0.49)*, P=*.47
12 wk	41.51±6.28	42.84±5.68	−0.61 (−1.18 to −0.04)*, P=*.04*
Within-group change (12wk)	−0.08 (0.57-0.63)*, P=*.59	0.09 (−0.31 to 0.48)*, P=*.67	
Hip flex-extension MIN			
Baseline	7.90±15.71	4.25±3.62	−0.23 (0.1-0.12)*, P=*.11
12 wk	4.45±5.26	6.32±5.53	−0.12 (0.39-0.45)*, P=*.41
Within-group change (12wk)	−0.11 (0.41-0.48)*, P=*.44	−0.19 (0.162-0.21)*, P=*.17	
Hip angle during heel strike			
Baseline	32.51±4.35	34.60±4.6	0.47 (−0.10 to 1.03)*, P=*.11
12 wk	38.96±4.83	36.13±3.57	−0.67 (1.22 to −0.09)*, P=*.02*
Within-group change (12wk)	−1.09 (−1.58 to −0.58)*, P>*.00*	−0.26 (−0.66 to 0.14)*, P=*.21	
Knee flex-extension MAX			
Baseline	61.76±18.43	60.44±9.05	−0.02 (0.86-0.88)*, P=*.86
12 wk	60.87±9.00	61.00±7.49	0.02 (−0.54 to 0.57)*, P=*.96
Within-group change (12wk)	−0.03 (0.83-0.87)*, P=*.84	−0.02 (0.912-0.944)*, P=*.91	
Knee flex-extension MIN			
Baseline	8.45±10.8	7.34±5.31	−0.13 (−0.69 to 0.43)*, P=*.65
12 wk	8.81±6.39	10.05±7.29	0.18 (−0.38 to 0.74)*, P=*.53
Within-group change (12wk)	−0.03 (−0.42 to 0.36)*, P=*.89	−0.4 (−0.8 to 0.02)*, P=*.06	
Knee angle during heel strike			
Baseline	7.86±5.11	7.63±2.06	−0.06 (−0.61 to 0.5)*, P=*.84
12 wk	8.14±4.02	7.52±2.23	−0.19 (−0.75 to 0.37)*, P=*.50
Within-group change (12wk)	−0.04 (−0.43 to 0.35)*, P=*.83	0.13 (−0.26 to 0.52)*, P=*.52	
Ankle dorsi-plantarflex MAX			
Baseline	21.83±3.13	20.37±4.87	−0.36 (−0.91 to 0.20)*, P=*.21
12 wk	16.18±3.39	18.86±4.9	0.63 (0.06-1.20)*, P=*.03*
Within-group change (12wk)	1.82 (1.17-2.46) *P>*.00*	0.25 (−0.15 to 0.65)*, P=*.22	
Ankle dorsi-plantarflex MIN			
Baseline	10.57±3.00	10.18±3.50	−0.12 (−0.67 to 0.44)*, P=*.67
12 wk	9.38±2.10	10.04±3.23	0.24 (−0.32 to 0.80)*, P=*.40
Within-group change (12wk)	0.40 (−0.11 to 0.80)*, P=*.06	0.06 (−0.34 to 0.45)*, P=*.77	
Ankle angle during heel strike			
Baseline	13.43±2.56	14.03±2.39	−2.24 (−0.32 to −0.80)*, P=*.40
12 wk	11.02±2.53	12.75±3.41	0.57 (0.01-1.12)*, P=*.04*
Within-group change (12wk)	0.91 (0.44-1.38)*, P>*.00*	0.47 (0.05-0.88)*, P=*.03*	

NOTE. MAX and MIN indicate the maximum and minimum joint angles across the gait cycle (0%-100%). For the ankle, MAX represents peak dorsiflexion and MIN represents peak plantarflexion.

⁎ indicates that there is a statistically significant difference between groups, *P*<.05.

### Balance function

Static balance outcomes did not differ between groups at baseline or at 12 weeks (table 5). In contrast, the YBT composite score increased by 12.71 points in the TCC group (72.61±9.22 to 85.32±9.68) versus 2.21 points in controls (74.49±8.12 to 76.70±10.19), with a significant between-group difference at 12 weeks (*P*<.01).

**Table 5:** Comparison of postural control ability at baseline and 12 weeks in the experimental and control groups (mean±SD)

	Experimental Group (n=23)	Control Group (n=23)	Between-Group Difference
Eyes open			
Trajectory length			
Baseline	697.93±34.99	703.58±47.68	−0.05 (0.69-0.75)*, P=*.71
12 wk	688.89±40.29	708.23±41.77	0.47 (−0.09 to 1.03)*, P=*.10
Within-group change (12wk)	0.15 (0.27-0.33)*, P=*.29	−0.13 (−0.53 to 0.26)*, P=*.51	
Trajectory area			
Baseline	299.40±38.06	304.02±35.94	0.13 (−0.43 to 0.68)*, P=*.66
12 wk	300.24±39.19	303.49±33.65	0.09 (−0.47 to 0.64)*, P=*.75
Within-group change (12wk)	−0.15 (−0.54 to 0.25)*, P=*.46	0.02 (−0.37 to 0.41)*, P=*.91	
Speed			
Baseline	14.35±0.56	14.65±0.82	0.44 (−0.13 to 1.00)*, P=*.13
12 wk	14.86±1.33	14.66±1.12	−0.17 (−0.72 to 0.39)*, P=*.55
Within-group change (12wk)	−0.38 (−0.78 to 0.03)*, P=*.07	0 (−0.40 to 0.39)*, P=*.98	
Eyes close			
Trajectory length			
Baseline	1140.15±42.78	1141.32±45.36	0.03 (−0.53 to 0.58)*, P=*.93
12 wk	1145.43±48.79	1151.13±53.71	0.11 (−0.44 to 0.67)*, P=*.70
Within-group change (12wk)	−0.39 (−0.79 to 0.02)*, P=*.06	−0.37 (−0.77 to 0.04)*, P=*.08	
Trajectory area			
Baseline	574.42±57.69	595.70±24.7	0.48 (−0.09 to 1.04)*, P=*.10
12 wk	594.32±36.79	600.14±26.89	0.18 (−0.38 to 0.74)*, P=*.53
Within-group change (12wk)	−0.34 (−0.74 to 0.07)*, P=*.10	−0.33 (−0.73 to 0.07)*, P=*.11	
Speed			
Baseline	20.65±0.68	21.03±0.84	0.49 (−0.08 to 1.05)*, P=*.09
12 wk	20.89±0.81	21.16±1.38	0.24 (−0.32 to 0.79)*, P=*.40
Within-group change (12wk)	−0.36 (−0.76 to 0.05)*, P=*.09	−0.12 (−0.51 to 0.28)*, P=*.55	
YBT			
Baseline	72.61±9.22	74.49±8.12	0.22 (−0.34 to 0.77)*, P=*.45
12 wk	85.32±9.68	76.7±10.19	−0.87 (−1.45 to −0.28), *P*<.01*
Within-group change (12wk)	−1.14 (−1.63 to −0.62), *P*<.01*	−0.24 (−0.63 to 0.16)*, P=*.25	

⁎ indicates that there is a statistically significant difference between groups, *P*<.05.

## Discussion

This trial suggests that a 12-week TCC program may improve gait and balance performance in patients with FAI. Compared with the health education control group, the TCC group demonstrated a narrower step width and favorable changes in selected sagittal-plane hip and ankle kinematics during walking, together with improved dynamic balance outcomes. These findings are consistent with potentially more stable or efficient walking strategies; however, mechanistic interpretations (eg, improved mediolateral control or increased confidence) should be regarded as hypothesis-generating because we did not assess neuromuscular mechanisms directly (eg, EMG or strength testing) and gait speed variability and task familiarization may also influence spatiotemporal and kinematic measures.

### Patient-reported ankle outcomes (pain and function)

A previous study reported that a 12-week TCC program improved CAIT scores in individuals with FAI, consistent with our findings.^22^ Relative to that prior work, the present trial additionally quantified spatiotemporal parameters and sagittal-plane hip, knee, and ankle kinematics using 3-dimensional motion capture and force plates, thereby providing more objective biomechanical insight into gait adaptations after TCC. We also observed that TCC training improved AOFAS scores and reduced VAS pain in patients with FAI. Chronic pain and ankle dysfunction can impose substantial physical and psychological burdens, ultimately limiting daily activities and quality of life. Prior studies have highlighted the broader benefits of TCC for chronic pain, including reductions in pain, stiffness, and functional limitations in osteoarthritis populations.^23^ By integrating mindful attention with slow, controlled movements, TCC may promote relaxation and facilitate pain modulation. In addition, TCC has been associated with improvements in balance, gait, muscle strength, and flexibility, as well as psychological factors such as confidence and attentiveness.^24^ In our cohort, pain relief may have also indirectly supported better perceived ankle function and walking ability.

Notably, the control group also demonstrated small improvements in CAIT, AOFAS, and VAS from baseline to 12 weeks. These changes may reflect the effects of the usual-care education provided (eg, advice on activity modification/rest, use of ankle bracing when necessary, and psychological support), which could reduce symptom provocation and improve perceived stability and pain. However, the magnitude of change in the control group did not reach the MCID thresholds for CAIT (≥3 points) or VAS (∼1.4 cm), and was below the AOFAS MCID estimates; therefore, these changes may have limited clinical relevance but should be considered when interpreting between-group effects.

### Gait biomechanics

The TCC group demonstrated a reduction in step width after 12 weeks. Step width reflects the mediolateral base of support and is commonly interpreted as a gait-stability strategy. In individuals with FAI, a wider base may be adopted to compensate for perceived instability.^25^ The narrowing of step width in the TCC group may indicate reduced reliance on a widened base of support strategy, although alternative explanations (eg, walking speed differences or learning effects) are possible. This finding should be interpreted cautiously and validated in future studies that control walking speed and include mechanistic measures.

Not all spatiotemporal variables changed significantly, which may relate to self-selected walking speed, sample size, and the possibility that TCC primarily influences balance-related strategies rather than temporal gait metrics. Future studies using standardized speeds, longer follow-up, or active comparators may help clarify whether additional spatiotemporal adaptations emerge.

Regarding sagittal kinematics, our results are reported as maxima/minima and angles at heel strike as defined in the Methods (eg, ankle dorsi/plantarflexion maximum represents peak dorsiflexion and minimum represents peak plantarflexion). In this context, the lower ankle dorsiflexion maximum and reduced dorsiflexion at heel strike observed after TCC may reflect a modified landing strategy and ankle-control pattern during early stance.^26^^,^^27^ However, without direct measures of joint stiffness, muscle activation, passive range of motion, or strength, mechanistic interpretation should remain cautious; small changes may also arise from speed variability or repeated-trial familiarization.

Similarly, changes in hip flexion should not be attributed solely to muscle strength. Hip kinematics may be influenced by flexibility, neuromuscular coordination, and multijoint compensation in response to distal impairments. Although TCC training may reduce movement-related fear and promote a less cautious gait strategy, this interpretation remains indirect in the absence of psychological or neuromuscular measures collected concurrently. Future work incorporating kinesiophobia measures, strength testing, and/or EMG could better determine the contributors to these kinematic changes and help differentiate training effects from learning or speed-related influences.

### Postural control adaptations

We observed improvements in dynamic stability as reflected by the YBT after 12 weeks. The TCC routine includes repeated single-leg support and transitional tasks (eg, controlled kicks), which may challenge lower-limb strength/endurance and sensorimotor integration in FAI individuals. TCC emphasizes coordinated control of head, trunk, and limb movements with continuous weight shifting, which may promote adaptive use of vestibular, visual, and proprioceptive inputs contributing to postural control.^28^

Prior studies have reported that TCC is associated with improved postural control across diverse populations.^29^ Improvements have been described in both static and dynamic balance, particularly in older adults, and have been linked with lower fall risk.^30^ The frequent weight transfer and multidirectional center-of-mass adjustments required during TCC may also support ankle proprioceptive function, including in individuals with sensory deficits.^31^ Although much of the available evidence comes from older-adult cohorts, emerging data suggest that TCC may also benefit dynamic balance and neuromuscular function in athletic populations with FAI, consistent with our findings and previous reports.^32^

### Study limitations

This study has several limitations. First, the single-center trial had a modest sample size, limiting generalizability. Second, the study was powered for CAIT, and AOFAS and VAS were secondary outcomes, not independently powered. Third, the intervention had an attention/contact-time imbalance (36 TCC sessions vs 3 brief education sessions), so findings mainly reflect TCC versus minimal care rather than an attention-matched active comparator. Fourth, gait analyses were limited to sagittal-plane kinematics, and multiplanar adaptations were not assessed. Fifth, mechanistic inferences are constrained by the lack of neuromuscular measures and the use of self-selected walking speed. Sixth, outcomes were measured immediately postintervention without longer-term follow-up, leaving durability uncertain. Future studies should include attention-matched active comparators, multiplanar biomechanics, and follow-up to evaluate sustainability and real-world delivery.

## Conclusions

A 12-week TCC program was associated with improvements in ankle instability/function and pain in patients with FAI, along with favorable changes in selected gait and balance outcomes. TCC may be a feasible, low-impact rehabilitation option for FAI; larger trials with longer follow-up are warranted.

## Suppliers

a. G*Power, version 3.1; Heinrich-Heine-Universität Düsseldorf.

b. SPSS, version 26.0; IBM.

c. ALPHA 2 heart rate monitor; Mio Global.

d. SMART-D 400 motion capture system (including BTS SMART software); BTS Bioengineering.

e. Huber; LPG Systems.

## Disclosure

The investigators have no financial or nonfinancial disclosures to make in relation to this project.

## Data statements

The datasets used and analyzed during this study are available from the corresponding author on reasonable request.

## Appendix Supplementary materials

Image, image 1 dummy alt text

Image, application 2

## Footnotes

Supplementary material associated with this article can be found, in the online version, at doi:10.1016/j.arrct.2026.100595.
