# Supplementary figures and images for: Effects of Tai Chi Chuan on Postural Stability and Lower-Limb Biomechanical Characteristics in Patients With Functional Ankle Instability: A Randomized Controlled Trial
Source: Arch Rehabil Res Clin Transl. 2026 Feb 4;8(2):100595. doi: 10.1016/j.arrct.2026.100595 (PMC13282805; doi:10.1016/j.arrct.2026.100595)

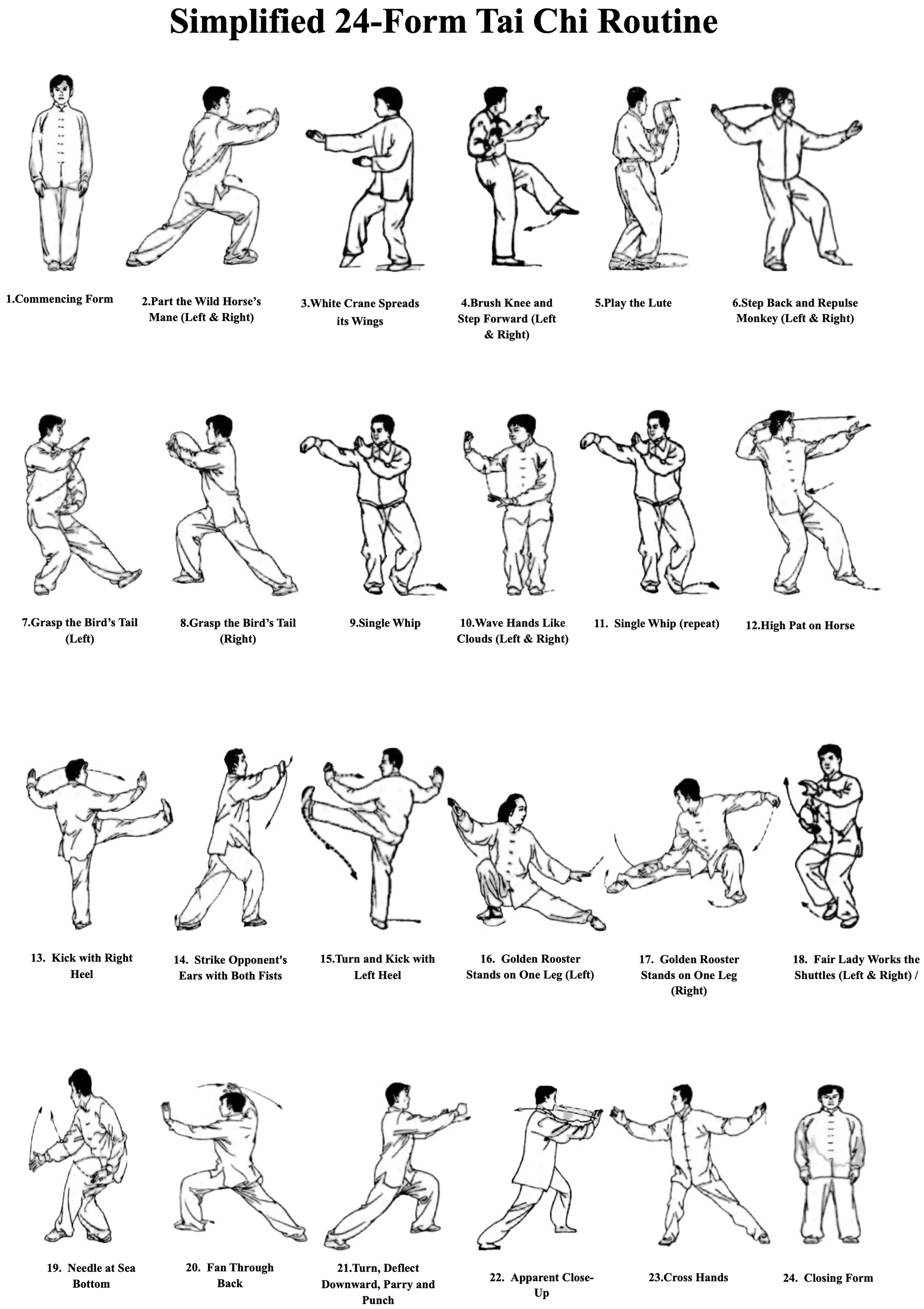

Supplement: Supplementary file 1 [file mmc1.jpg]
